# Alpha-Synuclein and Parkinson’s Disease Motor and Non-Motor Symptoms: What Is New?

**DOI:** 10.3390/life12060904

**Published:** 2022-06-16

**Authors:** Jessica Grigoletto, Emanuela Colla

**Affiliations:** Bio@SNS Laboratory, Scuola Normale Superiore, Piazza dei Cavalieri 7, 56126 Pisa, Italy; jessica.grigoletto@sns.it

Although it was discovered about 25 years ago, alpha-synuclein (αS) misfolding and accumulation in neuronal tissues is still recognized as one of the most crucial aspects in Parkinson’s disease (PD) pathology [1]. The recognition of non-motor symptoms as clinical signs of PD, supported by the appearance of Lewy bodies made of aggregated αS in extranigral areas, suggests a more ample and diffuse role of αS in PD pathogenesis. Therefore, this Special Issue aimed to reassess the missing link between a non-classical vision of PD and αS pathophysiology. A few major conclusions can be drawn.

(a) There is a strong impact of genetic variation on disease susceptibility, which involves accumulation of uncommon single nucleotide polymorphisms (SNPs) in the sequence of PD-associated genes, such as in αS, and which contributes to motor and non-motor symptom manifestation. As detailed by Magistrelli et al. [2], recurrent SNPs in PD-associated genes, their promoter sequence, or 3′-UTR may influence the appearance of non-motor symptoms, such as depression and cognitive decline, as shown in the case of Rep1 SNPs for αS. A detailed mechanism of toxicity has not been elucidated yet, but because of the nature of this modification, it is thought that recurrence of SNPs modifies disease susceptibility by acting on the related PD-associated gene’s expression level. In the case of αS, SNPs have been shown to cause accumulation of different spliced variants of its transcript, or as in the case of Rep1, the presence of this SNP seems to facilitate the recruitment of specific transcription factors or other DNA binding proteins;
(b) Cognitive decline and behavioral changes observed in PD patients [3] could be specifically associated with the dysregulation of opioid receptors’ expression level and a concomitant denervation of myelinated axons in the corticostriatal tracts, as shown in PD patients and murine models of α-synucleinopathy. As described by Grigoletto et al. [4], accumulation of extranigral αS aggregates in glutamatergic axonal bundles in corticostriatal WMTs is associated with a reduction in the expression of Mu-opioid receptors, a finding that was also confirmed in the brain of PD patients;
(c) Inflammation and neuroinflammation are a salient component of PD pathogenesis. In the CNS, astrocytes seem to play a major role in contributing to αS toxic function. As illustrated by Wang et al. [5], astrocytes could amplify propagation of toxic αS strains by acting as scavengers of αS aggregates, although astrocyte-to-neuron transmission seems to be less efficient than astrocyte-to-astrocyte. Alternatively, astrocytes could be directly activated by toxic αS through pattern recognition receptors (PRRs) and TLRs and induce neuroinflammation. Modulation of the immune response by αS point mutations has also been found in PD hereditary cases, with carriers showing a more pronounced production of proinflammatory cytokines [2]. Local peripheral inflammation has been described by us and others [6,7] as a key pathological step that is associated with the appearance of toxic αS. αS can stimulate local cytokine production by glia cells and macrophages. Still unknown is if and how this ability to elicit inflammation is related to the recently described function for αS to act as an antimicrobial peptide [8]. Interestingly, KO αS mice, which had been originally found to be healthy and with no significant neurological phenotype, presented greater susceptibility to morbidity when challenged by a viral infection [9,10]. Similarly, analysis of children’s intestinal biopsies after Norovirus infection showed a concomitant increase in intestinal αS that paralleled the level of inflammation [11]. While more evidence in vivo and in vitro is necessary to fully support this new function for αS, if proven, it could drastically expand our vision of this protein’s physiology.

Great advancements have been achieved in understanding the pathophysiology of PD and αS. Recognizing non-motor symptoms as an essential part of PD development has represented a step forward in our efforts to move toward a more global approach in treating PD. PD is now considered a multisystem illness, and αS represents a common target that could potentially unify the plethora of symptoms of PD patients. This Special Issue aims to serve as an initial step toward understanding the complexity of αS and toward offering new ideas or paths to pursue in a global effort to cure PD in the nearest future.
